# Evaluation of Spatial Relationships between Health and the Environment: The Rapid Inquiry Facility

**DOI:** 10.1289/ehp.0901849

**Published:** 2010-05-10

**Authors:** Linda Beale, Susan Hodgson, Juan Jose Abellan, Sam LeFevre, Lars Jarup

**Affiliations:** 1 Small Area Health Statistics Unit, MRC Centre for Environment and Health, Department of Epidemiology and Biostatistics, Imperial College London, United Kingdom; 2 Institute of Health and Society, Newcastle University, Newcastle Upon Tyne, United Kingdom; 3 CIBER Epidemiología y Salud Pública, Centre for Public Health Research, Valencia, Spain; 4 Bureau of Epidemiology, Utah Department of Health, Salt Lake City, Utah, USA

**Keywords:** disease mapping, environmental epidemiology, geographical information systems (GIS), risk analysis, spatial epidemiology, tool

## Abstract

**Background:**

The initiation of environmental public health tracking systems in the United States and the United Kingdom provided an opportunity to advance techniques and tools available for spatial epidemiological analysis integrating both health and environmental data.

**Objective:**

The Rapid Inquiry Facility (RIF) allows users to calculate adjusted and unadjusted standardized rates and risks. The RIF is embedded in ArcGIS so that further geographical information system (GIS) spatial functionality can be exploited or results can be exported to statistical packages for further tailored analyses where required. The RIF also links directly to several statistical packages and displays the results in the GIS.

**Methods:**

The value of the RIF is illustrated here with two case studies: risk of leukemia in areas surrounding oil refineries in the State of Utah (USA) and an analysis of the geographical variation of risk of esophageal cancer in relation to zinc cadmium sulfide exposure in Norwich (United Kingdom).

**Results:**

The risk analysis study in Utah did not suggest any evidence of increased relative risk of leukemia, multiple myeloma, or Hodgkin’s lymphoma in the populations around the five oil-refining facilities but did reveal an excess risk of non-Hodgkin’s lymphoma that might warrant further investigation. The disease-mapping study in Norwich did not reveal any areas with higher relative risks of esophageal cancer common to both males and females, suggesting that a common geographically determined exposure was unlikely to be influencing cancer risk in the area.

**Conclusion:**

The RIF offers a tool that allows epidemiologists to quickly carry out ecological environmental epidemiological analysis such as risk assessment or disease mapping.

Recent interests in developing environmental public health tracking systems, particularly in the United States and the United Kingdom, have provided an opportunity to advance techniques for spatial epidemiological analysis. The U.S. Centers for Disease Control and Prevention (CDC) and the U.K. Small Area Health Statistics Unit (SAHSU) have collaborated to adapt and enhance a tool for evaluating spatial relationships between health and environmental risk factors, for use in CDC’s National Environmental Public Health Tracking (EPHT) program (CDC 2004).

Since 2002, the CDC’s EPHT program has worked with local and state environment and health agencies, federal agencies such as the U.S. Environmental Protection Agency (EPA), academic institutions, and other nongovernmental agencies to develop and enhance the ongoing collection, integration, analysis, and interpretation of environmental hazards, human exposures to environmental hazards, and noninfectious health effects potentially related to these exposures (McGeehin et al. 2004). The key functions of the EPHT program are *a*) to compile a core set of nationally consistent health and environmental data and measures; *b*) discover, describe, exchange, analyze, and manage data; *c*) provide access to tools for managing and analyzing the data; and *d*) disseminate environmental public health information to the public (CDC 2006). As part of this coordinated network to develop tools and methods, a partnership between the EPHT network and the SAHSU was initiated in 2005.

The SAHSU was established in 1987 after a recommendation of the Black (1980) inquiry into the incidence of leukemia among children and young adults near the Sellafield nuclear plant. One of the main aims of SAHSU is to assess the risk to the health of the population from environmental factors, with an emphasis on the use and interpretation of routine health data. A tool, the Rapid Inquiry Facility (RIF), was developed at SAHSU to help staff respond rapidly, with expert advice, to ad hoc queries from funding departments about unusual clusters of disease, particularly in the neighborhood of putative environmental hazard sources (Aylin et al. 1999).

This early version of the RIF was designed specifically for use with SAHSU data. The RIF software and the processes behind it were then evaluated on a European level as part of the European Health and Environment Information System for Exposure and Disease Mapping and Risk Assessment Project (EUROHEIS and EUROHEIS 2), with several EUROHEIS partners making changes to the RIF to facilitate its use with data from other European countries (e.g., Ferrándiz et al. 2004; Juhász et al. 2010). With the commencement of the CDC EPHT, the RIF was seen as a valuable tool that, with further enhancement, could be used more widely, such as in U.S. state health departments, to facilitate the process of responding to environmental health issues from policy makers and the public alike.

This article describes the development of this spatial epidemiological tool and reports on two case studies that used the system.

The investigation of possible health effects around point sources of environmental pollution have traditionally been costly and time-consuming because health and population data relevant to the area under study would need to be assembled and analyzed ad hoc. Linking health, population, and exposure data allows environmental public health practitioners to evaluate the spatial and temporal relations between environmental factors and health (McGeehin et al. 2004). Using geographical analysis, researchers can assess spatial trends in rates and risks at different geographical scales and potential clusters of disease and ascertain areas of rapid population change or areas of good or poor health.

The contribution that geographical information science can make to spatial epidemiology is increasingly being recognized (Elliott and Wartenberg 2004; Jarup 2004). Ever growing numbers of epidemiological institutions and departments use geographical information systems (GIS), but health and population data sets used are not yet routinely stored in a GIS; instead, they are simply imported on an ad hoc basis. The ability to undertake spatial analysis without the need for significant data manipulation offers tremendous time advantages.

The RIF has been developed primarily to assess the links among environmental exposures, health outcomes, and risk for groups, or ecological-level analysis using readily available aggregated health and population data such as those collected for censuses. There are a number of inherent issues with ecological analysis, which include problems with data availability and suitability, as well as methodological challenges such as dealing with inconsistent geographical boundaries, different data scales, exposure misclassification, and ecological bias (Elliott and Savitz 2008). A number of different statistical methods can be used to estimate area-level risks and confidence intervals to give an assessment of associated uncertainty, which differ in suitability depending on the study (Beale et al. 2008). The methods included within the RIF aim to minimize many of the limitations indicated above, whereas exploiting the advantages offered by high-resolution data for large areas such as states or countries, and over long periods of time.

## Materials and Methods

The RIF has been described in detail elsewhere (Beale et al. 2008). Briefly, this tool is embedded in a GIS that requires ArcGIS (version 9.3; ESRI, Redlands, CA, USA) and connects to an external database such as Microsoft Access or Oracle of geocoded health and population data. This tight-coupled approach between a GIS and a database removes the need to explicitly gather data by study, saving on both time and data storage.

The RIF calculates directly standardized rates and indirectly standardized rate ratios in a user-defined population. Users can specify, via various means of selection or by spatial analyses, a “study population,” such as the population living within a certain distance of a point or area source of pollution or the populations of administrative areas falling within the jurisdiction of a health authority. All specified health events that occurred during the selected time period and that fall within this study area are retrieved, together with the relevant population data. The RIF can handle any health coding [e.g., *International Classification of Diseases* (ICD); ICD-Oncology; Surveillance, Epidemiology and End Results (SEER); and user-defined codes]; for data coded using the ICD-9 [World Health Organization (WHO) 2004] and ICD-10 (WHO 2007), the RIF provides lists to enable health events to be selected by chapters, groups, or individual codes. Applying the study population sex- and age-specific disease rates to the reference region population generates directly standardized rates. Indirectly standardized relative risks project the sex- and age-specific rates of the reference population to the age and sex structure of the study population producing standardized mortality ratios (SMRs) or standardized incidence ratios (SIRs). When defining the study area, users should take into account that the number of observed cases required to support valid SIRs are less than those required to support directly age-adjusted rates. In addition to sex and age, standardization by other covariate(s), such as socioeconomic status (SES), ethnicity, or income (where such data are available), is easily achieved.

Two main types of analysis can be carried out using the RIF: risk analysis and disease mapping. Using risk analysis, associations between either proximity (to point, line, or area sources) or exposure to a putative risk factor and health can be explored. Rates and relative risks are calculated in user-defined distance bands around one or more sources, or levels of exposure if such data are available. Users can also run homogeneity and linear trend tests to check if the risk is statistically homogeneous across bands. Disease mapping allows visualization of mortality or morbidity rates and spatial patterns of health at a user-defined geographical resolution. Maps are produced of rates and relative risks, including smoothed (toward the global mean) relative risks, by empirical Bayesian estimation using the Poisson-gamma model suggested by Clayton and Kaldor (1987). The RIF can also link with external software such as SaTScan (Kulldorff and Nagarwalla 1995) to search for disease clusters as well as WinBUGS (Bayesian inference using Gibbs sampling; Lunn et al. 2000) and integrated nested Laplace approximation (Rue et al. 2009) to produce smoothed (toward the local mean) risks based on the fully Bayesian model proposed by Besag et al. (1991). In all cases the results are mapped in the GIS.

Data can be easily exported from the RIF so that numerator, denominator, rates, and risks can be used elsewhere meaning the RIF adds functionality to the “epidemiologists’ toolkit” rather than replace existing tools or approaches. For example, users can export data to MS EXCEL, WinBUGS (Lunn et al. 2000), and SaTScan (Kulldorff and Nagarwalla 1995). In addition, the RIF can seamlessly generate reports in, for example, MS Word using XML-structured data (text).

The following sections illustrate the use of the RIF with two case studies: a risk analysis from the United States and a disease-mapping analysis from the United Kingdom.

## Results

### Case study: U.S. risk analysis

The Utah Department of Health began investigating a perceived excess of leukemia after requests from the local community at Woods Cross.

**Figure 1:**
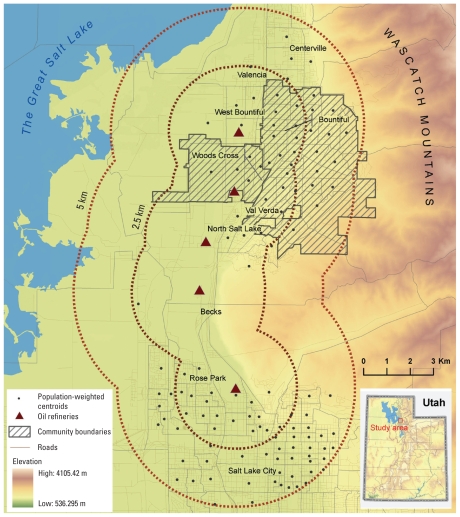
Oil refineries located near Woods Cross and Bountiful, Utah.

Oil refining activities in Utah started as early as 1909, with approximately seven oil companies working in 25 wells near Mexican Hat, San Juan County (Figure 1). The first of five currently operating refineries, located nearly adjacent to each other along an 11-km north–south corridor, was built in 1932 (Harline 1963; Strack 2007). These five refinery facilities process approximately 61 million barrels annually (Isaacson 2005). Industries associated with oil refining are colocated with these refineries, and several National Priority List hazardous waste sites are found in the vicinity (U.S. EPA 2009)

In 2004, these five refineries reportedly released 161,000 kg of hazardous air emissions, including benzene, cyclohexane, ethylbenzene, and ethylene. In addition, there are likely to be substantial fugitive releases of volatile organic hydrocarbon compounds from the transportation of oil by tanker truck and pipeline, from the transfer and processing of crude oil, and from the storage of product, and refineries release a number of processing-related air contaminants (U.S. EPA 2009). Leukemia, multiple myeloma, Hodgkin’s lymphoma, and non-Hodgkin’s lymphoma (NHL) have been associated with hazardous air emissions released by oil refineries (Infante 1993; Rushton 1993; San Sebastián et al. 2001; Smith et al. 2007).

Because it was not possible to accurately identify which populations were exposed, a pragmatic decision was made to assess risk in the census blocks within 2.5 km and between 2.5 and 5 km of the refineries. These distances capture sufficiently sizable populations for meaningful analysis, reflect differences in the topography of the area that may influence exposure, and conform to an earlier initial investigation, enabling comparisons to be made. The topography of Davis County forces the communities into a narrow band (~ 10 km wide running north to south). The communities are bounded by the steep Wasatch Mountain Range on the east and the Great Salt Lake on the west. Approximately 2.5 km east of the refinery, the topography changes (due to Lake Bonneville, an ancient parent inland sea), whereas at 2.5 km west of the refinery the land use transitions from mixed suburban and agricultural use to solely agricultural use. Bountiful is approximately 5 km south from the refineries at a higher elevation, suggesting that it may be at risk from plumes from stack emissions, particularly given that the predominant wind direction is southerly. Exposures at Woods Cross, within 2.5 km, are more likely a result of fugitive emissions because it sits in the shadow of the plume. Approximately 62,000 and 87,000 residents live within 2.5 km and between 2.5 and 5.0 km from the refineries, respectively.

Cancer incidence data on first primary leukemia (ICD-9 codes 204–207; ICD-10 codes C91–C95), multiple myeloma (ICD-9 code 203, ICD-10 code C90), Hodgkin’s lymphoma (ICD-9 code 201; ICD-10 code C81), and NHL (ICD-9 codes 200, 202; ICD-10 codes C82–C85) among Utah residents from 1973 to 2006 were obtained from the Utah Cancer Registry and investigated at census-block-group level. The cancer data were georeferenced by the Utah Environmental Public Health Tracking Network (UEPHTN), with > 98% being georeferenced to census block groups using case report residential address. Median income from the 2000 Census was used to control for confounding by SES. The latency for these cancers is generally assumed to be about 5 years, although longer latency periods have been reported (Crump and Allen 1984).

### Risk of leukemia, multiple myeloma, Hodgkin’s lymphoma, and NHL (SIR)

Cancer risks for populations of census blocks falling within 2.5 km of the five facilities (encompassing the residential area of Woods Cross) and between 2.5 and 5.0 km (capturing all surrounding communities) were compared with cancer risks for the State of Utah. Analysis was carried out, using the RIF, with 33 years of cancer incidence data to ensure stable estimates (Table 1).

Only the risks of NHL were statistically higher than would be expected given the reference rates of Utah. The values showed that males living closest to the refineries had the highest risk. SIRs adjusted for age and income were virtually the same as SIRs adjusted for age only, suggesting that the excess risks of NHL within 2.5 km of the oil refineries are not related to income. The socioeconomic structure of the population in Bountiful has changed during the study period, so analyses were also carried out using income from 1990, but again, income had little to no effect on the overall results.

Exposures are hard to establish, and although refinery boundaries have remained pretty stable during the past 30 years, the locations of stacks and sources of fugitive emissions have changed as plants have changed processes and/or built additional facilities and as ancillary industries around these sites have changed in use or been abandoned or relocated over time (with some becoming Superfund sites). The distance bands used in this study, although thoughtfully chosen, remain arbitrary boundaries.

These analyses did not show any evidence of increased risk of leukemia, multiple myeloma, or Hodgkin’s lymphoma in the populations around these five facilities. An excess risk of NHL was found in populations living around the oil refineries, and exposure to emissions from these sites is a possible explanation [e.g., Steinmaus et al. (2008) linked both leukemia and certain types of NHL to benzene exposure]. An excess of disease among men could also point to occupational exposure, although a substantial number of the refinery employees lived outside of the study area (e.g., north Davis County and Salt Lake County). Further investigation to identify what specific disease subtype is occurring and whether environmental or occupational exposures have contributed to risk would need to be conducted.

The above example shows how the RIF can help effectively use limited public health resources by identifying target populations who could most benefit from public health intervention, education, and early screening clinics.

### Case study: U.K. disease mapping

During the period of the “cold war” (1953–1964), the British Ministry of Defense undertook an extensive program of field trials to simulate the dissemination of toxic biological agents across the country (Academy of Medical Sciences 1999). The field trials involved the release of zinc cadmium sulfide (ZnCdS) from static devices, vehicles, aircraft, and ships. An independent review of these U.K. trials concluded that exposure to cadmium from the dissemination of ZnCdS during the cold war should not have resulted in adverse health effects in the population (Elliott et al. 2002). However, in 2005, a local surgeon suggested that there was an increased risk of esophageal cancer in Norwich caused by exposure of the local population to ZnCdS released by the Ministry of Defense in 1963 (Reacher et al. 2006).

The U.K. Health Protection Agency (HPA) carried out a preliminary investigation to assess whether there was any evidence that the residents of the city of Norwich, County of Norfolk, were at an increased risk of esophageal cancer during the period 1984–2003. Although no evidence was found that incidence rates were higher in Norwich than in the rest of England and Wales, they did find a higher incidence of esophageal cancer registrations in the County of Norfolk (Reacher et al. 2006). There were, however, some limitations to this work; in particular, rates and risks were not adjusted for age, sex, or SES. Therefore, a second study was recommended and was carried out using the RIF.

Incidence and mortality of esophageal cancer (ICD-9 code 150, ICD-10 code C15) for 1984–2003 (the same period as investigated by the HPA) were investigated at standard table (ST) ward level for Norfolk. Wards or electoral divisions are key elements of U.K. administrative geography and refer to the spatial units used to elect local government councilors. There are 7,932 ST wards in England, with an average population of 6,200. It was not possible to establish precisely which areas or populations were exposed to ZnCdS, but at least one trial involved dispersion of ZnCdS over Norwich (Academy of Medical Sciences 1999); thus, the population of Norwich could reasonably be identified as a potentially “exposed” group.

Indirect SIRs and SMRs were calculated taking the population of England and Wales as a reference, and risks were adjusted for age, sex, and SES. Population data from each decennial census (1981, 1991, 2001) were used, with populations interpolated for intercensal years using data on population growth. Adjustment for SES was by the Carstairs index (in quintiles), the 1991 census measure of deprivation (Carstairs and Morris 1990), which combines data on male unemployment, car access, social class, and overcrowding to allow geographical areas to be placed on a five-point scale from least to most deprived.

### Risk of esophageal cancer: SIR and SMR

Relative risks of esophageal cancer incidence and mortality were not higher in the City of Norwich than expected. After adjustment for SES, risks were statistically significantly lower in women than would be expected (Table 2). Directly standardized incidence and mortality rates per 100,000 person-years for Norwich were at the lower end of the range of those for England, reported to be 12.7–14.4 per 100,000 person-years among males and 8.6–9.3 per 100,000 among females for esophageal cancer incidence (during 1991–1999), and 14.4 per 100,000 among males and 8.2 per 100,000 among females for esophageal cancer mortality (during 1998) (Office for National Statistics 1999a, 1999b).

**Figure 2:**
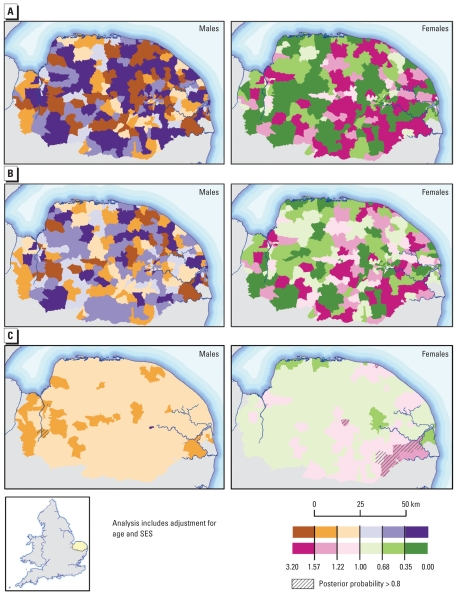
Risks of esophageal cancer incidence, Norfolk, United Kingdom, 1984–2003. (*A*) SIR. (*B*) SIR using empirical Bayes smoothing. (*C*) SIR using full Bayes smoothing (adjusted for age and SES). All analyses used England and Wales as a reference.

When adjusted relative risks of esophageal cancer incidence were mapped by ward across the County of Norfolk, no consistent patterns emerged (Figure 2). The areas of highest relative risk for males and females differed, suggesting a common geographically determined exposure (e.g., ZnCdS) was unlikely to be influencing esophageal cancer risk in the area. Table 3 shows the indirectly standardized risks across Norfolk.

The RIF performs empirical Bayes smoothing of the raw relative risks to account for sampling variability in the observed data (Figure 2B). Full Bayesian smoothing was also carried out by direct linkage to WinBUGS (Lunn et al. 2000) (Figure 2C). This approach allows computation of a measure of uncertainty, the posterior probability of an excess risk (Pr[RR > 1 | data]), associated with the smoothed risk values (Richardson et al. 2004). These exceedence probabilities (Figure 2C) are also displayed in the RIF (Beale et al. 2008). Areas with relative risks > 1 and posterior probabilities > 0.8 are areas that we are 80% confident of an excess relative risk. Analogously, areas with relative risk and posterior probability < 0.2 have a low relative risk with a probability of 80%. The impact of the adopted approach upon results can be clearly seen in Figure 2. The empirical Bayes model smoothes the risk toward the global mean, without accounting for spatial autocorrelation; hence, the geographical pattern is similar to that of the raw SIRs, although with less extreme relative risks. The full Bayes model smoothes risks locally, thus accounting for spatial autocorrelation, so the risk pattern is less similar to those produced by the other two approaches, and the smoothing effect is more noticeable.

The routinely collected data on cancer registrations and mortality did not provide evidence of an increased risk of esophageal cancer incidence and mortality in the population of Norwich or Norfolk compared with England and Wales. The disease-mapping analysis did not reveal any areas with higher relative risks common to both males and females.

## Discussion

Health and population data are increasingly becoming available; however, these data tend to be aggregated to administrative geographies due to the nature of data collection and/or confidentiality issues. The use of aggregated data in epidemiology has associated problems; nonetheless, ecological studies can be useful for detecting associations between exposure distributions and disease and can help target resources for further individual research. Several statistical methods can be used to estimate area-level rates and risks that can be mapped to aid interpretation. However, the availability of statistical techniques and tools to calculate and map small area risks does not necessarily ensure that meaningful results are achieved. All methods will be reliant on sufficient, accurate, and complete underlying health and population data, as well as appropriate interpretation. Biases, ecological or otherwise, can be introduced into any spatial epidemiological study, and the methods used in the RIF are no exception. More details of limitations of spatial analysis are presented elsewhere (Beale et al. 2008) and are touched on below.

When undertaking a risk analysis, the study area should be selected to represent a population exposed (or perceived to be) to the pollution source or pollutant of interest. Accurately identifying the “at risk” population is crucial and attempts should be made to reduce exposure misclassification when, for example, using proxy variables or indirect exposure assessment (Hodgson et al. 2007). Several different methods for defining areas of exposure have been incorporated into the RIF, including various methods to select specific areas geographically and spatially. Distance from source can be used as a proxy for exposure, although it can be difficult to define what distances reflect a meaningful exposure differential. Ideally factors such as prevailing wind, topography, and emissions should be taken into account. A wide variety of GIS methods and bespoke dispersion models (for discussions, see Elliott et al. 2000; Jerrett et al 2005) are now available to improve pollution modeling and exposure assessment, and data from these packages can be imported into the RIF to more accurately represent areas of exposure. However, no matter how well modeled or how appropriately monitored, environmental levels do not necessarily equate to exposure (Briggs 2003), and ecological data will not represent individual exposure.

Ecological data do not offer the spatial accuracy that individual data afford, and in addition to defining areas of exposure, effective selection of any affected populations is crucial. In ecological analyses, the total populations of an administrative region will be classed as exposed if that region falls within the defined exposure area. However, using more complex spatial analysis, as permitted with GIS, users can reduce any “overselection”of population by including areas based on the geometric centroid (in terms of its shape) or, better still, by using population-weighted centroid data. Both geometric and population weighted centroids can be used to select potential at risk populations in the RIF, although only geometric centroids are dynamically calculated at runtime. Of course, all GIS functions for spatial selection are available to users, or indeed, they can specify the study population using prior knowledge.

Aggregated data tend to be collected for administrative areas that can change over time, causing problems for long-term studies. Further health and population data are often collected for administrative areas (e.g., census regions), whereas environmental data will often be collected or modeled, for different often nonconformable spatial areas. Indeed, data are often needed for geographical zones that do not correspond to administrative boundaries, and effectively combining data with different spatial boundaries takes care and skill to minimize errors than can be introduced at this stage (Briggs et al. 2008). The scale of analysis will affect the results obtained. Indeed, results will be, in part, a consequence of the chosen resolution of the data because of the “modifiable areal unit problem” (Openshaw 1984), where changes in spatial aggregation or arrangement of areas can affect the underlying values for those areas, so observations are usually only relevant for the scale of analysis. To avoid the ecological fallacy, associations observed in an ecological study should not be imputed to individuals.

Attention must be given to the latency between exposure and disease onset to ensure the appropriate period of health data is used. Populations can change over time, including between censuses, so as study years extend from a census year, population estimates can become increasing unreliable. Interpolating between the census years can improve values, but nevertheless, this can be a source of error. Indeed, errors or variations in small-area population counts can create major uncertainties, especially where health events are rare. In addition, for conditions with even a relatively short lag period, but especially for those end points with lag periods of decades, such as solid tumors, the impact of population migration must be considered. Over time, people will move in and out of the study area, or between exposure categories. In many cases, lack of suitable data precludes effectively accounting for migration, but failure to take migration into account, particularly where migration rates are known to be high, can result in exposure misclassification, biased risk estimates, and reduced study power, with the extent of migration bias related to both the magnitude and direction of the migration (Tong 2000). There is a need to further develop and use suitable methods to deal with this bias in spatial epidemiological analysis and tools, such as the RIF.

For some health end points, such as hospital admissions, the ascertainment of data can differ between areas with local variations, introducing spurious spatial variation where higher disease incidence might be due to specialist local centers, proactive general practitioners, local disease registers, or local screening programs (Hansell et al. 2001). Conversely, lower disease incidence might be due to rurality or boundary effects, with populations moving between administrative areas to seek treatment. Furthermore, changes in the recording of health data, for example, changes between ICD revisions, can introduce spurious temporal and/or spatial variation (Anderson and Rosenberg 2003).

The RIF affords numerous time and efficiency savings compared with an ad hoc, study-by-study approach; however, the same care and consideration should be put into designing a RIF query as would be put into any other approach. This includes appropriate consideration for exposure assessment, study population identification, choice of appropriate comparison population, mapping resolution, interpretation, and so forth.

## Conclusion

The RIF provides a valuable tool for initial analyses of environmental health problems, providing ecological risk estimates indicating the presence or absence of a public health problem. The RIF cannot be used to assess individual-level associations or causal relationships, although evidence of an association observed from a RIF study will provide support for undertaking a more costly individual-level study that may provide stronger evidence of causality. Methods such as those provided by the RIF allow investigative studies to be carried out across large geographical areas and over long time periods, avoiding constraining any analysis to a low number of cases, thereby increasing the statistical power to estimate the excess risk (Olsen et al. 1996). Such studies may otherwise be too involved to provide timely responses to any public concerns.

The RIF is one of several tools that support EPHT work and is designed to complement already existing methods. The RIF is distributed as freeware with documentation (Beale et al. 2007), upon application to the CDC EPHT program (CDC 2004) or SAHSU (2009).

## Figures and Tables

**Table 1:** Indirectly standardized risks for leukemia, multiple myeloma, Hodgkin’s lymphoma, and NHL around the five refinery facilities, Utah (1973–2006).

	Distance > 2.5 km					Distance of 0–2.5 km				
		Adjusted for age		Adjusted for age and income			Adjusted for age		Adjusted for age and income	
Health end point/sex	Observed	Expected	SIR (95% CI)	Expected	SIR (95% CI)	Observed	Expected	SIR (95% CI)	Expected	SIR (95% CI)
Leukemia										
Male	103	101.88	1.01 (0.83–1.23)	103.52	1.00 (0.82–1.21)	145	140.46	1.03 (0.88–1.21)	140.82	1.03 (0.87–1.21)
Female	80	79.63	1.00 (0.80–1.25)	79.88	1.00 (0.79–1.25)	100	106.96	0.93 (0.76–1.14)	108.08	0.93 (0.75–1.13)
Male + female	183	181.50	1.01 (0.87–1.17)	183.40	1.00 (0.86–1.15)	245	247.42	0.99 (0.87–1.12)	248.91	0.98 (0.87–1.12)

Multiple myeloma										
Male	34	36.84	0.92 (0.64–1.29)	36.99	0.92 (0.64–1.28)	52	50.69	1.03 (0.77–1.35)	50.07	1.04 (0.78–1.36)
Female	34	30.73	1.11 (0.77–1.55)	30.93	1.10 (0.76–1.54)	35	41.70	0.84 (0.58–1.17)	40.48	0.86 (0.60–1.20)
Male + female	68	67.57	1.01 (0.78–1.28)	67.92	1.00 (0.78–1.27)	87	92.39	0.94 (0.75–1.16)	90.54	0.96 (0.77–1.19)

Hodgkin’s lymphoma										
Male	26	25.42	1.02 (0.67–1.50)	26.70	0.97 (0.64–1.43)	34	37.49	0.91 (0.63–1.27)	37.68	0.90 (0.62–1.26)
Female	27	21.29	1.27 (0.84–1.85)	21.45	1.26 (0.83–1.83)	30	29.55	1.02 (0.68–1.45)	31.85	0.94 (0.64–1.34)
Male + female	53	46.71	1.13 (0.85–1.48)	48.15	1.10 (0.82–1.44)	64	67.04	0.95 (0.74–1.22)	69.52	0.92 (0.71–1.18)

NHL										
Male	148	118.23	1.25 (1.07–1.47)	119.45	1.24 (1.05–1.46)	184	165.75	1.11 (0.96–1.28)	165.75	1.11 (0.96–1.28)
Female	118	108.30	1.09 (0.91–1.31)	107.88	1.09 (0.91–1.31)	141	147.21	0.96 (0.81–1.13)	146.90	0.96 (0.81–1.13)
Male + female	266	226.53	1.17 (1.04–1.32)	227.32	1.17 (1.04–1.32)	325	312.96	1.04 (0.93–1.16)	312.65	1.04 (0.93–1.16)

CI, confidence interval.

**Table 2:** Indirectly and directly standardized rates of incidence and mortality from esophageal cancer in the City of Norwich (1984–2003), using the population of England and Wales as a reference.

	Incidence					Mortality				
		Adjusted for age		Adjusted for age and SES			Adjusted for age		Adjusted for age and SES	
Sex	Observed	Expected	SIR (95% CI)	Expected	SIR (95% CI)	Observed	Expected	SIR (95% CI)	Expected	SIR (95% CI)
Indirectly standardized risks										
Male	159	165.03	0.96 (0.82–1.13)	173.8	0.91 (0.78–1.07)	166	162.09	1.02 (0.88–1.19)	169.41	0.98 (0.84–1.14)
Female	87	120.92	0.72 (0.58–0.89)	125.19	0.69 (0.56–0.86)	85	112.63	0.75 (0.60–0.93)	116.04	0.73 (0.59–0.91)
Male + female	246	285.95	0.86 (0.76–0.97)	298.99	0.82 (0.73–0.93)	166	162.09	1.02 (0.81–1.03)	285.46	0.88 (0.78–1.00)
	Observed	Rate (per 100,000 person-years)		Rate (per 100,000 person-years)		Observed	Rate (per 100,000 person-years)		Rate (per 100,000 person-years)	

Directly standardized risks										
Male	159	13.20 (11.30–15.44)		11.47 (9.38–14.03)		166	13.68 (11.74–15.94)		12.68 (10.12–15.90)	
Female	87	6.25 (5.00–7.72)		6.09 (4.52–7.94)		85	6.07 (4.84–7.52)		5.79 (4.28–7.57)	
Male + female	246	9.64 (8.5–10.93)		8.71 (7.41–10.24)		251	9.78 (8.63–11.07)		9.15 (7.67–10.91)	

CI, confidence interval.

**Table 3:** Indirectly standardized risks of esophageal cancer in the County of Norfolk (1984–2003), using the population of England and Wales as a reference.

		Adjusted for age		Adjusted for age and SES	
Sex	Observed	Expected	SIR (95% CI)	Expected	SIR (95% CI)
Male	1,181	1239.33	0.95 (0.90–1.01)	1215.53	0.97 (0.92–1.03)
Female	736	792.43	0.93 (0.86–1.00)	780.99	0.94 (0.88–1.01)
Male + female	1,917	2031.76	0.94 (0.90–0.99)	1996.52	0.96 (0.92–1.00)

CI, confidence interval.
